# An Unusual Case of Mitral Stenosis with Coronary Artery and Left Ventricular Apical Aneurysm

**Published:** 2014-01-12

**Authors:** Mohammad Masoumi, Maryam Aliramezany

**Affiliations:** 1 *Physiology Research Center, Kerman University of Medical Sciences, Kerman, Iran.*; 2 *Research Center for Health Services Management, Institute of Futures Studies in Health, Kerman University of Medical Sciences, Kerman, Iran.*

**Keywords:** *Heart ventricles* • *Coronary vessels* • *Aneurysm* • *Mitral valve stenosis*

## Abstract

The left ventricular aneurysm is one of the most significant complications of myocardial infarction and is defined as the expansion of the ventricular wall. The coronary artery aneurysm is characterized by an abnormal dilation of the localized portion of the coronary artery, and its main cause is atherosclerosis. We herein report an unusual case of coronary artery and left ventricular aneurysms in a mitral stenosis patient with normal coronary arteries and no sign of atherosclerosis. This patient was a known case of mitral stenosis due to rheumatic heart disease and was symptomatic despite optimal medical therapy. Laboratory tests were normal, and electrocardiography showed sinus rhythm and left atrium abnormality without pathologic Q wave. Angiographic view illustrated left circumflex artery and left ventricular apical aneurysms. Percutaneous transvenous mitral commissurotomy was performed successfully and the patient was discharged with Warfarin and a beta blocker. No symptom was observed at six months' follow-up.

## Introduction

The left ventricular (LV) aneurysm is one of the most important complications of myocardial infarction and is thought to develop in 5-10% of all patients with acute myocardial infarcion.^1^ The LV aneurysm, defined as the expansion of the dyskinetic area of the LV wall, is generally allied to myocardial infarction or coronary malformation, although it can also be associated with other rare causes. The coronary artery aneurysm is characterized by an abnormal dilation of a localized portion of the coronary artery^2^^, ^^3^ and is typically diagnosed incidentally on coronary angiography. Small coronary aneurysms have been observed in up to 5% of patients undergoing coronary angiography.^2^

The main causes of the coronary artery aneurysm in the world are atherosclerosis, congenital origin, and mycotic-embolic disease.^4^ Here we present a case of the LV and coronary artery aneurysms in a mitral stenosis patient with normal coronary arteries.

## Case Report

A 60-year-old woman with a previous history of rheumatic heart disease and mitral stenosis was referred for percutaneous transvenous mitral commissurotomy (PTMC). Despite optimal medical therapy, she was symptomatic. The laboratory findings were within normal limits: blood sugar of 81mg/dl; triglyceride of 152mg/dl, cholesterol of 151mg/dl; high-density lipoprotein of 46mg/dl; low-density lipoprotein of 74mg/dl; blood urea nitrogen of 44mg/dl; and creatinine of 0.9mg/dl.

Electrocardiography revealed sinus rhythm and left atrial enlargement without pathological Q-waves. Transthoracic echocardiography illustrated normal LV size, ejection fraction of 50%, apical dyskinesia, moderate to severe mitral stenosis, mild aortic regurgitation, and pulmonary artery pressure of 50 mmHg. The patient underwent invasive coronary angiography and ventriculography, which confirmed the aneurysmal dilatation of the left circumflex artery and the LV apical aneurysm. There was no stenosis in the epicardial coronary artery. PTMC was performed successfully, and the patient was discharged with Warfarin and a beta blocker. The patient was followed up three and six months after discharge. She did not report any complaint, and echocardiography only indicated LV aneurysm without any change in its size. Additionally, pulmonary artery pressure was normal and no gradient was observed on the mitral valve.

## Discussion

The LV aneurysm mainly occurs after myocardial infarction, especially at the anterior or apical segment belonging to the territory of the left anterior descending artery.^5^ The incidence of the LV aneurysm is reported to be about 5-10% of all patients with acute myocardial infarction.^6^ In one study, the LV aneurysm was quite rare, with a prevalence of 0.76% in 12,271 consecutive adult patients undergoing cardiac catheterization.^7^

The LV aneurysm can be either congenital or acquired. Congenital LV aneurysms are rare and lethal, while acquired LV aneurysms have either a cardiac or a non-cardiac etiology. The most frequent cardiac causes of the LV aneurysm is myocardial infarction, but this kind of aneurysm can also occur in hypertrophic cardiomyopathy, dilated cardiomyopathy, sarcoidosis, arrhythmogenic right ventricular dysplasia, myocarditis, glycogen storage disease, Chagas disease, and hyper-immunoglobulin E syndrome.^6^^, ^^8^ Patients with the LV aneurysm may be either asymptomatic or symptomatic manifesting with recurrent arterial emboli, angina, congestive heart failure, ventricular tachycardia, or sudden cardiac death.^9^ The indication for surgery is only symptomatic LV aneurysms, which are -in order of frequency- comprised of angina, congestive heart failure, and ventricular tachycardia.

The coronary artery aneurysm is an uncommon disease and is defined as a coronary artery dilation (either saccular or fusiform) which can exceed the diameter of the normal adjacent segment by 1.5-2 times.^10^ Atherosclerotic lesions account for the majority of the causes of the coronary artery aneurysm. Other etiologies include congenital dissection, infection, vasculitis, post coronary intervention lesions, and other inflammatory lesions. The etiology may vary according to the geographic location (Kawasaki disease in Japan and atherosclerosis in North America).^11^ The right coronary artery, followed by the left anterior descending artery, is the most frequently involved artery.^11^ Most atheromatous aneurysms are small, have a thick wall, and pose a low risk of spontaneous rupture.^2^ Congenital aneurysms are unusually found in the right coronary artery. This kind of aneurysm is generally large and is most commonly found in young patients.^12^ The coronary artery aneurysm can be asymptomatic or complicated by thrombosis or embolization with subsequent ischemia and rupture.^11^ The optimal therapy for patients with the coronary artery aneurysm is unknown and controversy persists regarding the use of medical or surgical modalities for symptomatic coronary artery aneurysms. Be that as it may, most authors agree that surgery should be reserved for patients with significant coronary stenosis or those with significant angina despite adequate medical treatment. 

Our mitral stenosis patient had LV and coronary artery aneurysms despite having normal coronary arteries. It is noteworthy that LV aneurysm formation in patients with normal coronary arteries has been rarely reported elsewhere. Coronary angiography revealed no significant stenosis in the coronary arteries, which excluded the possibility of ischemic heart disease. The other etiologies of LV and coronary artery aneurysms can usually be ruled out by history taking, clinical examination, and laboratory tests. The aneurysms of the coronary artery and the LV are usually correlated with atherosclerosis and commonly thought to be caused by atherosclerosis; nevertheless, they may represent the superimposition of autoimmune diseases or inflammatory processes. Atherosclerosis is a common cause of LV and coronary artery aneurysms in adults, but only 1.5% of patients with atherosclerosis have aneurysms^13^ and this small group may be composed of those who are genetically susceptible.

## Conclusion

The presence of the LV and coronary artery aneurysms in our mitral stenosis patient, who had normal coronary arteries, hints at the possible role of inflammation and rheumatic heart disease. Our literature search yielded no reported case of the LV and coronary artery aneurysms associated with mitral stenosis and normal coronary arteries. In our case, the absence of underlying stenotic atherosclerotic lesions in the coronary artery involved, as well as the absence of symptoms attributed to the LV and coronary artery aneurysms, prompted us to follow up our patient conservatively without surgical intervention.

## Notes

***This paper should be cited as: ***Masoumi M, Aliramezany M. An Unusual Case of Mitral Stenosis with Coronary artery and left Ventricular Apical Aneurysm. J Teh Univ Heart Ctr 2014;9(1):*43-45.*

## References

[1] Altay H, Altin C, Coner A, Muderrisoglu H (2011). Normal coronary artery patient presenting with left ventricular aneurysm. Case Rep Med.

[2] Swaye PS, Fisher LD, Litwin P, Vignola PA, Judkins MP, Kemp HG, Mudd JG, Gosselin AJ (1983). Aneurysmal coronary artery disease. Circulation.

[3] Robinson FC (1985). Aneurysms of the coronary arteries. Am Heart J.

[4] Daoud As, Pankin D, Tulgan H, Florentin Ra (1963). Aneurysms of the coronary artery. Report of ten cases and review of literature. Am J Cardiol.

[5] Antman EM, Braunwald E, Braunwald E (2011). ST segment elevation myocardial infarction: management. Heart Disease: A Textbook of Cardiovascular Medicine.

[6] Kao PF, Hung WY, Ko WC, Yang HY, Chan P (2005). Left ventricular aneurysm with normal coronary angiogram: a case report and literature review. Acta cardial sin.

[7] Ohlow MA, Secknus MA, Geller JC, von Korn H, Lauer B (2009). Prevalence and outcome of congenital left ventricular aneurysms and diverticula in an adult population. Cardiology.

[8] Paul M, Schäfers M, Grude M, Reinke F, Juergens KU, Fischbach R, Schober O, Breithardt G, Wichter T (2006). Idiopathic left ventricular aneurysm and sudden cardiac death in young adults. Europace.

[9] Paraskevaidis S, Stavropoulos G, Vassilikos V, Chatzizisis YS, Polymeropoulos K, Ziakas A, Dakos G, Parcharidis GE (2009). Idiopathic left ventricular aneurysm causing ventricular tachycardia with 1:1 ventriculoatrial conduction and intermittent wenckebach block. Open Cardiovasc Med J.

[10] Syed M, Lesch M (1997). Coronary artery aneurysm: a review. Prog Cardiovasc Dis.

[11] Assiri AS (2000). Giant coronary artery aneurysm. Ann Saudi Med.

[12] Konen E, Feinberg MS, Morag B, Guetta V, Shinfeld A, Smolinsky A, Rozenman J (2001). Giant right coronary aneurysm: CT angiographic and echocardiographic findings. AJR Am J Roentgenol.

[13] Nichols L, Lagana S, Parwani A (2008). Coronary artery aneurysm: a review and hypothesis regarding etiology. Arch Pathol Lab Med.

